# Advancements in Cold-Region Rice Breeding: The 4S Phenotypic-Design Breeding System and Its Applications in Heilongjiang

**DOI:** 10.3390/plants13182658

**Published:** 2024-09-23

**Authors:** Baohai Liu, Shoujun Nie, Shiwei Gao, Qing Liu, Yuqiang Liu, Fengchen Mu, Bo Zhang, Beiping Zhao, Hongru Gao, Licheng Wu, Minggang Xiao, Kun Li

**Affiliations:** 1 Quality and Safety Institute of Agricultural Products, Heilongjiang Academy of Agricultural Sciences, Harbin 150086, China; swslbh@163.com; 2 Suihua Branch of Heilongjiang Academy of Agricultural Sciences, Suihua 152052, China; nsj-0821@163.com (S.N.); gaoshiwei1118@126.com (S.G.); liuqing@haas.cn (Q.L.); liuyuqiang@haas.cn (Y.L.); 3 Biotechnology Research Institute, Heilongjiang Academy of Agricultural Sciences/Heilongjiang Rice Molecular Breeding Engineering and Technology Research Center, Harbin 150028, China; mfc888221@163.com (F.M.); zhangbo222@sina.com (B.Z.); 13945094556@139.com (B.Z.); 13766959996@139.com (H.G.); yezi_320@163.com (L.W.); likun.steven@163.com (K.L.); 4 Heilongjiang Province Key Laboratory of Crop and Livestock Molecular Breeding, Harbin 150028, China

**Keywords:** rice, phenotype, 4S phenotypic breeding system, breeding technology

## Abstract

Heilongjiang, located in the cold region, is China’s largest rice-producing and commercializing province. The variety selection of rice in cold regions (RCR) is indispensable in promoting the rice industry’s development, and technological innovation in breeding theory plays a significant role in breeding breakthrough. Based primarily on long-term research, contemplation, and breeding practices, the 4S (selected topic, selected breeding, selected progenies, and selected promotion) phenotypic-design breeding technical system for RCR, which has revolutionized the theoretical basis and deepened the conceptual model, has been developed through systematic analyses and generalization. The system covers several key aspects such as scientific question formulation, breeding objective optimization, parental taxa hybridization, progeny population selection, and innovation dissemination, aiming to improve the foresight, precision, and efficiency of breeding. Furthermore, the system has demonstrated successful cases of rice variety breeding over the past 20 years, such as for Suijing 3, Suijing 4, Suijing 18, and a series of other rice varieties, providing theoretical and technical supports for cold-region rice breeding.

## 1. Introduction

The rice area of Heilongjiang Province, located in the north of China, is the world’s northernmost cold rice-planting area, and has distinct ecological characteristics [1]. The region has a clear ecological particularity, with an annual active accumulated temperature of more than 10 °C, ranging from 1900 to 2700 °C. This region can be divided into six ecological temperature zones, suitable for the cultivation of early-maturing varieties with insensitivity to light and temperature [1]. As its main grain crop, in Heilongjiang, the rice commodity amount in 2020 was of 27.029 million tons, accounting for 77.4% of the northeast region and 20.3% of the whole country’s rice production, with a rice commodity rate of 93.3%, ranking first in China [2]. According to statistical data, 954 new rice varieties have been authorized from 1949 to 2022 [3]. These varieties have evolved significantly in terms of yield, quality, and resistance, establishing a rice germplasm resource population with cold-region characteristics, which have been crucial to rice production throughout history [1,4,5,6,7,8]. Furthermore, these varieties strategically guarantee food security [9], significantly influencing the national economy and people’s livelihoods [10].

Rice breeding involves the development of varieties with new or improved traits [11]. These processes require germplasm mining and utilization and the innovative application of breeding technology. To breed excellent novel rice varieties in cold regions (RCR), breeders have continuously explored effective methods based on different breeding theories. Several theories, such as the Northern Japonica Rice Ear-Type Improvement [12], Cold-Region High-Yield Strain Characteristic “Three Highs” Breeding [6], Cold-Region Early Japonica Rice “One-Early and Three-Resistant” New Strain Breeding [13], and Cold-Region Semi-Upright and Density-Resistant Early Japonica Rice Design [14], have been developed. These theories have guided breeding practices and therefore have produced numerous excellent rice varieties. In recent years, bio-breeding technologies, especially molecular design, gene editing, genome-wide selection, and artificial intelligence breeding, have rapidly developed in the emerging frontier interdisciplinary field of variety breeding methods. These technologies have achieved gene identification, modification, and aggregation, as well as the prediction of complex traits. However, no matter what kind of new technology it is, it is a tool and means to serve breeding, and, in the end, it must return to the basic logic of plant growth [15]. Bio-breeding, as a strong supplement to conventional breeding, requires the collaborative support of conventional breeding techniques. Compared with the quantity and quality of RCR variety selective breeding over the past decades, the innovation capacity of the breeding theory remains insufficient, lacking key innovations and limiting the selective breeding of innovative varieties. To further improve the foresight, precision, and efficiency of RCR breeding, we tested the hypothesis that breeding breakthrough rice varieties is the ultimate goal of 4S (selected topic, selected breeding, selected progenies, and selected promotion) phenotypic-design breeding for RCR. In addition, a practical factual traceability approach was adopted to systematically analyze the conventional 4S phenotypic-breeding technical system and empirically demonstrate the selective breeding process of rice varieties. This technology system, integrated with bio-breeding techniques, can effectively regulate the external characteristics of crops and germplasm resources, enhance the efficiency of selection and breeding, and achieve sustainable development in agriculture.

## 2. Construction of 4S Phenotypic-Design Breeding Technical System for RCR

### 2.1. Theoretical Foundations of the Technical System

The RCR phenotypic-design breeding technical system theory assumes the existence of high-yielding, high-quality, multi-resistant breakthrough rice varieties. RCR 4S phenotypic-design breeding aims to cultivate breakthrough large varieties. Rice breeding involves selecting for organisms with consistently innovative or improved traits and variety superiority, consistency, and stability. Since rice domestication approximately 10,000 years ago, breeding techniques have been used for successfully breeding new varieties. These techniques included hybrid breeding in the middle to late 19th century, traditional breeding in the late 19th and early 20th century, and molecular breeding in the 1980s. The development of these techniques has evolved from initial phenotype-based selection to the current genome- or gene-level design, representing a gradual process of advancement and technologization. Rice 4S phenotypic-design breeding follows Mendel’s plant genetics law. Furthermore, the genealogical method of hybrid breeding technology was used to select new varieties using rice phenotypic trait data statistics, precision optimization, and breeding practices, which purposefully perform artificial parental hybrid grouping and individual selection of hybrid progeny to breed new varieties, thus improving breeding foresight, precision, and efficiency. The RCR 4S phenotypic-design breeding technical system is based on the unique ecological environment of Heilongjiang Province’s rice-planting area and the realistic needs of its selectively bred varieties. This system is based on innovative technical concepts integrated and condensed at the intersection of crop breeding, crop cultivation, ecology, germplasm resources, statistics, and hydraulic engineering. After over 20 years of efforts, the Suijing 3, Suijing 4, and Suijing 18 rice varieties were developed.

### 2.2. Conceptual Model of the Technical System

**Figure 1:**
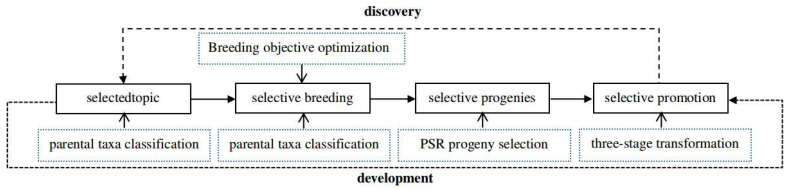
The 4S conceptual model for phenotypic-design breeding in RCR.

Based on the hypothesis that the ultimate goal is to cultivate breakthrough rice varieties, adhering to the concept of high-quality and sustainable development in rice breeding which integrates selected topics, selective breeding, selective progenies, and selective promotion together, research was conducted on models involving scientific question formulation, breeding objective optimization, parental taxa hybridization, progeny population selection, and innovative dissemination. This led to the creation of the 4S conceptual model for phenotypic-design breeding in RCR with the aim of improving the foresight, precision, and effectiveness of breeding (Figure 1).

### 2.3. Construction of Technical System Connotation

#### 2.3.1. Techniques for Formulating Key Scientific Questions in Breeding

**Figure 2:**
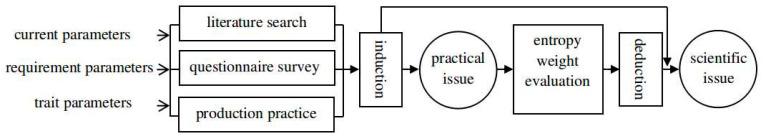
Conceptual model of the methodology for formulating scientific questions in RCR breeding.

A literature search, questionnaire survey, production practice, entropy evaluation, and induction and deduction were used to formulate key scientific breeding questions from the explanation of production practice questions and to satisfy the need for the innovation of excellent RCR variety traits (Figure 2). Subsequently, a multi-dimensional search for RCR breeding strategies was conducted to comprehensively and objectively present issue information. Additionally, the essential logical relationship between rice breeding practices and scientific issues was determined using several thinking inquiries, such as practicing, recognizing, and re-practicing specific problems. A rational understanding of the issues of breeding was obtained, characterized by specificity and generalization, thereby guiding RCR’s novel variety selective breeding technology.

#### 2.3.2. Multi-Objective Optimal Design Techniques for Breeding Innovation

Based on the genetic algorithm and entropy weight evaluation method, the multi-objective optimal design was aimed at the breeding requirements of cold-region rice for the next 5 to 10 years [16]. It addressed the issue that the current variety’s yield and quality traits were contradictory and not easily balanced, as well as the difficulty in quantifying and coordinating these traits. Additionally, it tackled the problems in practical breeding processes where there was a predominance of experiential qualitative assessments over objective quantitative analyses, and a lack of coordination due to an overemphasis on single traits rather than a unified approach. Using the 22 agronomic traits of 7 main rice varieties promoted in the second thermal zone of Heilongjiang Province as a reference, a multi-objective optimization function model was constructed. The NSGA-II genetic algorithm and the entropy weight comprehensive evaluation method were applied to optimize and design a rational scheme for the target trait breeding parameter values that meet the needs of the region. This has improved the theory and methods of target breeding design for cold-region rice, avoiding subjective evaluation errors compared to traditional experience-based and single quantitative breeding strategies. This novel approach achieved a coordinated design of breeding objectives that were not easy to balance, and was highly scientific, universal, and practical for long-cycle rice breeding practices. The technology could be used for the scientific and quantitative design of multi-objective breeding for new rice varieties in different cold-region ecological zones, thereby improving breeding efficiency.

#### 2.3.3. Innovations in Parental Group Division and Crossing for Breeding

Based on the screening and group division techniques of Heilongjiang Japonica rice’s breeding parents [17], 16 agronomic and quality trait data (taste, brown rice rate, head rice rate, chalky grain rate, chalkiness, gel consistency, amylose content, active accumulated temperature, plant height, panicle length, panicle grain number, thousand-grain weight, yield, leaf blast, neck blast, and spikelet sterility rate) of 36 Japonica rice varieties dominantly popularized on a large scale in Heilongjiang in 2017 were systematically classified using principal component, cluster, and discriminant analysis methods to specify 9 principal component traits and factors, namely, active accumulated temperature, yield, gelatin consistency, amylose content, head rice rate, neck blast, thousand-grain weight, spikelet sterility rate, and brown rice rate. The discriminant function Y_i_ = a_1_X_1_ + a_2_X_2_ + a_3_X_3_ + a_4_X_4_ + a_5_X_5_ + a_6_X_6_ + a_7_X_7_ + a_8_X_8_ + a_9_X_9_ + c was established (where parameter a corresponds to the coefficients of each trait, variable X corresponds to the nine principal component traits, and c corresponds to the constant), successfully classifying the 36 main Japonica rice varieties into four groups. The percentage of crossbred varieties selected by parental mating between neighboring groups was 53.0%, with 29.4% between intermediate groups, and 17.6% between members of the same group, clarifying the order of parent selection in future breeding practices as between neighboring groups, between intermediate groups, and within the same group. Varieties within the same group have a high degree of similarity in all considered traits, while adjacent groups have similarities in some traits and close scores in the principal component analysis, but still sufficiently distinguishable as different groups. Intermediate groups may have differences in some traits but also have some similarities in other traits, depending on their positions in the feature space. Furthermore, 170 Japonica rice varieties authorized in Heilongjiang from 2005 to 2016 were divided into groups. This process overcame the limitations of intuitive and empirical classification by individual traits alone and provided reliable theoretical and data support for systematic group delineation and discrimination of other Japonica rice variety resources in cold regions. These methods can guide the rational selection and use of the parental resources of different groups in Japonica rice breeding, effectively improving the success rate of new variety selection and the effect of large-area promotion.

#### 2.3.4. Innovations in Pressure–State–Response (PSR) Selection and Realization Techniques for Hybrid Progeny

**Figure 3:**
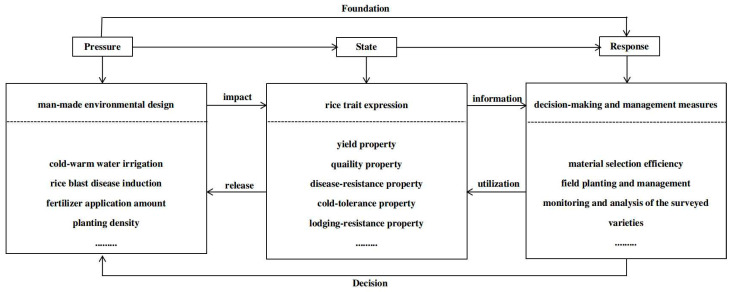
Pressure–state–response (PSR) conceptual model for hybrid progeny selection in Heilongjiang Japonica rice.

The PSR conceptual model for selecting cold-region Japonica rice hybrid progeny was developed using the sustainable development concept of pressure-based, trait-reflective, response-guided decision-making [18] (Figure 3). The hybridization breeding genealogy was used to select RCR hybrid progeny (F_2_–F_n_ generation) using the dynamic evaluation index system. In particular, an artificial dynamic stress environment was designed to collect trait evaluation indexes and implement management decision measures based on trait heritability in each hybridization generation. An evaluation system with one objective, three criteria, and eighteen indicators was developed. Subsequently, a combination of objective entropy weighting and efficacy scoring was employed for comprehensive index evaluation. The results indicated that the indexes of resistance to panicle blast, lodging level, and the rate of empty grains were the most critical considerations in the selection of hybrid offspring in cold-region rice breeding. This technology effectively overcame the difficulties in aggregation, identification, and selection efficiency caused by multiple factors, such as relying solely on breeding experience, not combining qualitative and quantitative characteristics, focusing on trait selection, and not systematically responding to decision-making. It realized an efficient aggregation and objective selection and evaluation of multi-optimal traits in hybrid progeny that is feasible, practical, efficient, and operable and produces a more reasonable selection scheme for hybrid progeny in RCR breeding.

#### 2.3.5. Development of Three-Stage Technology for the Efficient Transformation and Promotion of Scientific and Technological Achievements

**Figure 4:**
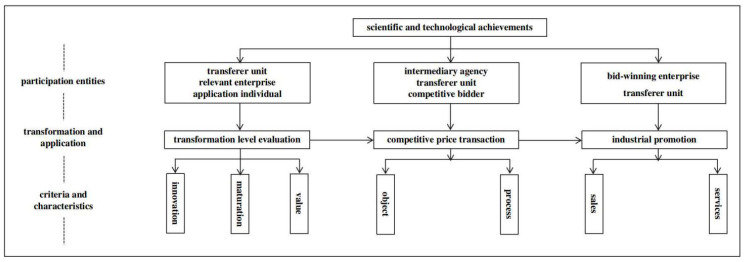
Conceptual model of a multidisciplinary, three-stage transformation of scientific and technological achievements.

Based on the sustainable development concept of the efficient transformation of scientific and technological achievements and a win–win situation for participating subjects, a three-stage scientific and technological achievement transformation conceptual model (Figure 4) was established using “1 + N” multiple subjects. The conceptual model includes transformation level evaluation, competitive price transaction, and industrial promotion. The transformation level evaluation is led by an achievement transfer unit, with the participation of multiple related enterprises and individual users, to evaluate the innovation, maturity, and commercial value of the achievements in terms of technical levels; the competitive price transactions are led by an achievement transformation transaction intermediary institution, with a transfer unit and bidding unit jointly participating to implement competitive price transactions according to the processes of audit, acceptance, and evaluation; and the industrial promotion is led by the winning enterprise with achievements that implement the industrialization and promotion of scientific and technological achievements, with full-process scientific and technological services such as personnel training and technical problem-solving provided by an achievement transfer unit.

In the process of transforming and promoting the achievements of science and technology, to reveal the regularity of the price evaluation of the first transformation and the second transfer of scientific and technological achievements, the PCI (Price Evaluation Method of Comprehensive Index Value) (Formula (1)) and PIC (Price Evaluation Method of Income Contribution Rate) (Formula (2)) mathematical decision-making models were created. These models were comprehensive and generalizable, providing decision support for the reasonable pricing of scientific and technological achievements, and could be referred to and promoted. Furthermore, to coordinate the design of the target characteristics, criterion characteristics, and index characteristics of the scientific and technological achievements, a result evaluation index system with 3 innovation, 8 maturity, and 5 commercial value indicators, totaling 16 index characteristics, was established. Among them, innovation referred to the scientific and technological content and advancement of the variety, mainly including variable indicators such as yield (x_1_), head rice rate (x_2_), and taste score (x_3_); maturity referred to the stability of the variety in trial cultivation, mainly including indicators such as lodging level (x_4_), empty shell rate (x_5_), panicle neck blast index (x_6_), brown rice rate (x_7_), chalky rice rate (x_8_), chalkiness (x_9_), gel consistency (x_10_), and amylose content (x_11_); and commercial value referred to the market monetary price of the variety’s application, mainly including indicators such as adaptation area (x_12_), intellectual property rights (x_13_), economic benefits (x_14_), social benefits (x_15_), and market environment (x_16_). This evaluation system achieved a scientific overall design and an objective presentation of index value information, avoiding the possibility of subjective evaluation errors, and the evaluation results were true and reliable, with strong feasibility and practicality.

In addition, a “packaging” platform bidding and trading process for scientific and technological achievements and technical services was constructed. In particular, a one-time payment of funds for transforming achievements could be used to meet the requirements of the subject group’s income and unit expenditures. The annual reproduction technical service fee guaranteed the funding requirements for in-depth innovation of the subject groups, thus realizing the sustainable development of scientific and technological achievements. The technical tracking service method was used following the transformation of scientific and technological achievements. This innovative initiative solved the problem that the business subject was affected by a lack of technical skills, partial understanding of achievement characteristics, and inadequate supervision of the supporting technology by realizing the synergistic effect of “1 + 1 > 2”. The three-stage technology application of scientific and technological achievement transformation and promotion through the participation of multiple subjects effectively avoided the overestimation of achievement transformation units, substantially reduced business risks caused by the transformee’s inability to objectively grasp the characteristics of the achievement level, guaranteed the fairness and competitiveness of the transaction process, and realized good docking between innovative achievements and their practical applications.

(1) $$P=P_{a}\times \frac{\lambda_{t}\sum {CI}_{i}(t)}{\lambda_{a}\sum {CI}_{i}(a)}$$

In Equation (1), *P* is the appraisal price of the achievement to be transformed, *P_a_* is the recent market transaction price of similar achievements, *CI_i_ (t)* is the efficacy composite index value of the achievement to be transformed, *CI_i_ (a)* is the efficacy composite index value of similar achievements, *λ_t_* is the *CI_i_ (t)* weighted value, and *λ_a_* is the *CI_i_ (a)* weighted value.

(2) $${P^{\prime }=P\times (1+x\times k)}^{y-1}$$

In Equation (2), *P′* is the second proposed evaluation price of the same achievement, *P* is the evaluation price of the achievement to be transformed for the first time, *x* is the annual rate of return from operating the achievement, *k* is the contribution rate of the return of the achievement, and *y* is the time of transformation (*y* ≤ 5).

## 3. Application and Achievements of 4S Phenotypic Breeding Technical System for RCR

The RCR 4S phenotypic-design breeding technical system, which serves as a breeding guideline, is an innovative technical concept condensed from years of exploration into breeding research, work practices, and multidisciplinary cross-integration by several generations. More than 30 “down-to-earth” excellent rice varieties have been developed, improving the traditional conventional breeding effects through the organic combination of qualitative and quantitative design and the selection of rice phenotypic agronomic traits. Its representative varieties are described below.

### 3.1. Suijing 3

Suijing 3 [19], authorized in Heilongjiang Province in 1999, established a historical record planting area of over 267,000 hm^2^ of self-breeding authorized varieties in the province that year and was awarded the province’s third prize of Scientific and Technological Progress. In 2012, the Suijing series of rice varieties bred using the core germplasm of Suijing 3 won the Heilongjiang Provincial Scientific and Technological Progress’s second prize. Sixty-two rice varieties were bred with Suijing 3 as their parent from 2008 to 2022 [3]. Suijing 3’s selective breeding process focuses on field pressure selection, with the experimental site located in an “old rice field” region with a high incidence of rice blast disease, and which was irrigated with underground wells. Consequently, its disease resistance was significantly greater than that of the control variety, Hejiang 1. When Heilongjiang Province experienced an extremely rare cold disaster obstacle, the rice’s setting rate still exceeded 85%, demonstrating a strong resistance to cold. Furthermore, it has excellent lodging resistance, and live stalks mature normally without premature senescence. Overall, Suijing 3 represents a successful attempt by breeders to investigate field-pressure selection in innovative practice.

### 3.2. Suijing 4

Suijing 4 [20], authorized in 1999, is the first aromatic Japonica rice variety in Heilongjiang Province, overcoming the shortage of aromatic rice in this province. This variety was awarded the second prize for Heilongjiang’s Provincial Scientific and Technological Progress in 2005. With Suijing 4 as their parent, 122 rice varieties have been bred up until 2022 [3]. Suijing 4 is a crossbreeding achievement of Lianxiang 1/(zygote12-34-1) F_2_ as the mother and (Matsumae/Jinian 2) F_5_ as the father, aiming to select varieties with high quality and flavor, after 15 years of crossbreeding under the experimental environment of “old rice fields” with a high rice blast incidence and well-water irrigation. The variety received a taste rating of 24.0 from the Ministry of Agriculture Cereals and Products Quality Supervision, Inspection, and Testing Center (Harbin), China, with a distinct flavor, no chalky grains, and a glossy appearance. In addition, it exhibits a good cold-tolerance performance in the field. It has strong tillering, slight leaf and neck blast symptoms, high lodging resistance, large spikes, abundant grains, convergent plants, mature live stalks, and good productivity. The entire plant has a distinct flavor from seedling to maturity. The Suijing 4 variety is a successful attempt by breeders to meet the demand for high-quality aromatic rice by focusing on breeding objectives and field pressure to select successful varieties in innovative practices.

### 3.3. Suijing 10

Suijing 10 [21], authorized in Heilongjiang Province in 2008, won the third prize for Heilongjiang Provincial Science and Technology Progress in 2014. Suijing 10 was bred through crossbreeding genealogy, with Shangyu 397 as the mother and Suijing 3 as the father. Suijing 10 has excellent biological characteristics, scoring 82.3 points for taste. Its quality indexes meet the national standard of Grade 2 high-quality rice. Suijing 10 yielded 8341.1 kg/hm^2^ of rice, 10.4% higher than that of the control Dongnong 416. Its plant height is 92.3 cm, plant shape is convergent, leaf color is dark green, and live stalks are mature. Additionally, it exhibits strong lodging resistance, tillering, and blast resistance, with no field disease. In 2006–2007, its cold tolerance appraisal revealed an empty-grain rate of 6.25–8.34%, and its cold tolerance was superior to that of the control Dongnong 416. Suijing 10 inherited its maternal high quality, good taste, strong tillering capacity, and good cold tolerance, as well as its paternal high yield, lodging resistance, and high setting rate. Thus, Suijing 10 has the advantages of high quality, high yield, lodging resistance, disease resistance, and strong adaptability. The Suijing 10 variety is a successful example of breeders’ innovative practice for actively and purposefully selecting parents for hybridization.

### 3.4. Suijing 18

Suijing 18 [18,22], authorized in Heilongjiang Province in 2014, was awarded the first prize for Heilongjiang Provincial Scientific and Technological Progress in 2018 and for the National Scientific and Technological Progress in 2021. In 2018, Suijing 18 was planted in an approximately 682,000 hm^2^ area, making Suijing 18 the largest conventional Japonica variety in China in terms of area of popularization. In Suijing 18 selective breeding, in response to the main limiting factors affecting the high and stable yield of rice, such as low temperature, cold damage, rice blast, lodging, and the lack of high-quality fragrant Japonica varieties, the scientific question “What would a novel rice variety suitable for cultivation in the second cumulative temperature zone of Heilongjiang Province, with high quality and flavor, high and stable yield, cold, disease, and lodging resistance, be like?” was raised. Based on this question, the optimized breeding objectives were as follows: (1) quality: fragrant Japonica, exceeding the national “quality rice” standard above the second grade, good taste; (2) yield: the province’s regional trial fields yielded 5% more than the control varieties and Suijing 4, with an average yield of 8000 kg/hm^2^ or more; (3) maturity: focusing on the 12-leaf maturity suitable for planting in Heilongjiang Province’s second cumulative temperature zone; (4) cold resistance: cold damage-resistant test indicating a 20% empty-grain rate; (5) disease resistance: under three years of continuous inoculation, leaf blast ≤ grade 6, and ear and stem blast ≤ grade 5; (6) lodging resistance: appropriate reduction in plant height, developed root system, short basal internodes, and strong stalk toughness. In its parental hybridization, the mother, Suijing 4, and the father, Suijing 3, were classified as class II [17], belonging to the same germplasm taxa. The mother, Suijing 4, has lodging resistance, cold tolerance, and good flavor. The father, Suijing 3, has a high combining ability, high yield, and lodging and disease resistance. PSR technology was used on the hybrid progeny to establish a phenotypic trait evaluation system by designing increased field selection pressure. Breeders practiced discrimination and the efficacy of integrated decision-making to select practical, efficient, and operable hybrid progeny. The implementation of scientific and technological achievement level evaluation, bidding and trading, and industrial promotion of three-stage integration of technical content involves transformation units, associated enterprises, and application individuals. This measure established a new record of CNY 41 million for the continuous transformation of a single variety of rice in Heilongjiang Province, and bid-winners earned CNY 168 million of profit and CNY 5.74 billion in new societal benefits over the five-year transformation period. Suijing 18 is a successful example of breeders actively and purposefully integrating scientific issue selection, parental hybrid selection, PSR selection, and three-stage transformation selection and promotion technologies in their innovative practices.

### 3.5. Suijing 28

In 2018, Longjiang Province authorized Suijing 28 [23]. Its breeding objectives were set based on the following scientific question: “What would a new, early-maturing, high-quality, stress-resistant, aromatic rice variety look like?” (1) Quality: rice milling quality, appearance quality exceeding the national “high-quality rice” standard grade 1, with a taste score of ≥80 points; (2) yield: provincial regional and production test fields attain more than a 5% increase in yield over the control, with an average yield of 8000 kg/hm^2^ or more; (3) maturity: the number of leaves in the main stem is 12, and the active accumulated temperature is 2450–2500 °C; (4) stress resistance: the empty-grain rate in a 3-year cold damage resistance test is ≤15%, with leaf, ear, and stem blasts of ≤3; (5) lodging resistance: appropriate plant height, developed root system, strong stalk, high toughness, and excellent lodging resistance, suitable for mechanized harvest. For parental selection, the mother, Suijing 4, and the father, Suijing 11, were classified as class II and class I, respectively [17], belonging to the germplasm of neighboring taxa. The father’s variety was characterized by strong low-temperature, cold damage, and rice blast resistance. The hybrid progeny was subjected to PSR technology. Based on the three-stage transformation, selection, and promotion technology, Suijing 28 was transformed immediately after its authorization.

### 3.6. Longdao 363

Longdao 363 [24] was authorized in 2020. The variety requires a ≥10 ℃ active accumulated temperature of approximately 2750 °C for approximately 146 d from seedling to maturity in the adaptation area. It has a 14-leaf main stem, plant height of approximately 105 cm, stalk length of approximately 18 cm, long-grain appearance, number of grains per spike of approximately 130, and 1000-grain weight of approximately 27 g. Two-year quality analysis results suggest a husked rice rate of 80.9–82.1%, head rice rate of 67.2–69.1%, chalky grain rate of 3–4%, chalkiness degree of 0.7–1.0%, straight-chain amylose content (dry basis) of 17.0–17.1%, gelatin consistency of 77–80 mm, and taste quality of 81 points, exceeding the national “high-quality rice” standard of the second grade. Three-year inoculation for resistance appraisal suggests a leaf blast grade of 1–3 and an ear and stem blast grade of 0–3. A three-year cold resistance test suggests an empty-grain rate of 9.26–15.24%. Longdao 363 breeding and authorization was a successful attempt by breeders to purposefully select progeny with lodging and disease resistance.

### 3.7. Songkejing 108

Songkejing 108, a Japonica-type conventional aromatic rice variety nationally authorized in 2020, aims to solve the scientific problem of “What is the domestication method for introducing the southern Japonica lineage to Northeast cold-region Japonica rice?” Its mother, Wuxiangjing 9, is from the Jianghuai region of southern China and has good taste quality, a strong flavor, and disease and lodging resistance. However, under normal conditions in Heilongjiang, it cannot mature and be harvested. Its father, Songjing 6, from the first cumulative temperature zone of Heilongjiang, has good adaptability, strong tillering, excellent rice quality, and high stress resistance. The PSR conceptual model was used for hybrid progeny selection. Songkejing 108 inherited the following advantages of both parents: (1) early maturity: full-life span of 145.6 d, 0.1 d earlier than the control Longdao 20; (2) high quality: stronger flavor, 67.6% head rice rate, 3.2% chalkiness degree, 15.1% straight-chain starch content, gelatin consistency of 61 mm, alkali-spreading value of grade 6.8, and a length-to-width ratio of 2.2:1; (3) high yield: the average yield was 8197.50 kg/hm^2^ in the trial field in 2018, 3.90% higher than the control Longdao 20; (4) strong stress resistance: rice blast composite index of 3.9 and 1.8 in two years, highest ear and neck loss rate of grade 5, moderate susceptibility to rice blast, and setting rate of 85.1%. Songkejing 108 realized achievement transformation immediately after authorization, owing to the three-stage transformation selection and promotion technology.

### 3.8. Songkejing 110

Songkejing 110, a conventional rice variety in Heilongjiang Province, is the sister line of Songkejing 108, and its selective breeding method is the same as that of Songkejing 108. Songkejing 110 requires a ≥10 °C active cumulative temperature of approximately 2700 °C in a growing period of approximately 142 d. This variety has a 13-leaf main stem, plant height of approximately 100.5 cm, spike length of approximately 19.1 cm, long-grain type, number of grains per spike of approximately 120 grains, and 1000-grain weight of approximately 24.8 g. A two-year quality analysis suggests a husked rice rate of 80.5–81.3%, head rice rate of 66.4–67.6%, chalky grain rate of 2.0–3.0%, chalkiness degree of 0.2–0.4%, straight-chain amylose content (dry basis) of 18.30–18.79%, gelatin consistency of 74–80 mm, crude protein (dry basis) content of 6.57–8.46%, and taste quality of 82 points, exceeding the national “high-quality rice” standard of the second grade. Three-year inoculation for resistance appraisal suggests a leaf blast grade of 0–3 and an ear and stem blast grade of 0–3. A three-year cold-tolerance test suggests an empty-grain rate of 5.93–21.30%. Its 2018–2019 regional test field attained an average yield of 8293.6 kg/ha, a 5.1% increase compared to that of the control variety Longdao 18. The 2020 production test field yielded 8329.1 kg/hm^2^, a 6.9% increase compared to that of the control variety Longdao 18. Songkejing 110 was transformed immediately after its authorization.

## 4. Prospects for RCR 4S Phenotypic-Design Breeding Technical System

### 4.1. Technical Systems Require Continuous Maturation and Practice-Based Development

Different regions have different ecological conditions and variety selection requirements due to the ecological characteristics of RCR planting areas. Therefore, based on the characteristics of Heilongjiang’s six cumulative ecological temperature zones, the breeding target design should be optimized at the current rice breeding level based on subjective thinking, objective evaluation to rational understanding, production practices, and the dynamic condensation of scientific issues. For example, rice varieties grown in a cumulative temperature zone should have superior taste, disease and lodging resistance, head rice rate, and yield level. Rice varieties in the second cumulative temperature zone should have better yield, taste, and disease resistance. Furthermore, rice varieties grown in the third and fourth cumulative zones should have a better taste and cold and lodging tolerance. To increase breeding parent selection efficiency, new rice variety resources should be continuously organized and enriched. Hybridized progenies are suitable for creating stress conditions, investigating status indicators, and testing seeds over several generations. Adjustments can be made according to breeding objectives, practices, and other response indicator selections. Achievements can only be valued when they are transformed and promoted as products. Therefore, products and their supporting services are the lifeblood of enterprise development. To maximize the product value, the advantages of “production, academia, and research institutions” must be collected as a leading role and synergistic function, and an integrated high-quality promotion system of “research, production, processing, marketing, and service” must be created. In addition, the 4S breeding technology system can also be applied in the breeding of soybeans, corn, coarse grains, and other crops, and continuously refined and optimized to meet evolving breeding objectives and environmental conditions.

### 4.2. Conventional and Molecular Breeding Technologies Must Be Integrated

Since the 1980s, new molecular biology breeding techniques have been developed. Conventional crossbreeding techniques have advanced significantly since the mid-to-late 19th century. In many cases, breeding practices are more important than genetic theory. Breeders have achieved what geneticists deemed impossible. For countless years, geneticists have anticipated identifying high-yield genes and performing targeted introgression [25]. The conventional hybrid breeding technology is advantageous for Heilongjiang rice (96.2% of the total number of rice varieties from 1949 to 2021), serving as a facilitator and inspector for molecular breeding technology innovation and implementation. Meanwhile, conventional hybrid breeding technology should not be ignored now or in the future but rather strengthened and upgraded. Through molecular breeding technologies such as gene editing, whole-genome selection, transgenesis, cell engineering, allele mining, molecular assistance, and molecular design, the replication, transcription, translation, and expression of DNA base sequence genetic information among biological individuals are achieved, enabling the direct selection and effective aggregation of excellent micro-level genes, thus more precisely controlling excellent traits. After gaining a comprehensive and in-depth understanding of the genetic characteristics of target traits, parents with excellent traits and containing target genes could be selected, and conventional breeding techniques (such as hybridization, backcrossing, synthetic crossing, selfing, etc.) could be used for breeding [26]. Integrating molecular breeding techniques into the 4S breeding technology system and artificially creating suitable breeding conditions and environments through purposeful matching and selection allow the phenotypic traits controlled by genetics and variation genes to be fully developed in the natural environment, and, by carrying out qualitative and quantitative macro-comprehensive judgments and usage of phenotypic traits, to achieve the ever-changing breeding goals in different ecological regions and production conditions. Thus, conventional hybrid breeding, along with molecular breeding and other advanced technologies, should be continuously integrated and developed depending on the specific breeding conditions, such as personnel, financial, and material resources. This can break the small-team “all-round” big breeding mode, eliminate restrictions on excellent genetic resources, and solve the deadlock of insufficiently efficient combinations of existing technologies. In addition, this initiative can overcome the isolation of promotion units and establish a breeding technical system comprising practicable conventional hybrid breeding and highly efficient, replicable, and easy-to-propagate biotechnology breeding.

### 4.3. The Technology Must Be “Activated” during Continuous “Evolution”

Philosophical theories such as the laws of the unity of opposites, mutual change between quality and quantity, the negation of the negation, and dialectical materialism have effectively guided cotton breeding [27]. The strategy of “empirical turn” in the study of technology philosophy was adopted to analyze the details of traditional breeding and transgenic technologies, elucidating their characteristics and differences [25]. Breeding has developed from a science with art to an art with science. Breeding is a highly abstract and systematic philosophy [28]. Technology is a type of “positive–negative” intermittent “evolution” of the “living” body, necessitating both human subjective awareness of scientific and technological progress and natural evolution. Breeding technology involves pooling good genotypes or genotype collections. The practice of selecting and breeding biological populations with consistency, excellence, and stability is the field-specific materialization of technology, presenting technical attributes and breeding-specific characteristics. Self-revolution in response to social production needs can be realized by adhering to breeding theory and through the continuous recognition, generalization, and re-recognition of breeding practices.

## Data Availability

The data presented in this study are available on request from the corresponding author.
